# Identification of Methylated Genes Associated with Aggressive Clinicopathological Features in Mantle Cell Lymphoma

**DOI:** 10.1371/journal.pone.0019736

**Published:** 2011-05-16

**Authors:** Anna Enjuanes, Verònica Fernàndez, Luis Hernández, Alba Navarro, Sílvia Beà, Magda Pinyol, Armando López-Guillermo, Andreas Rosenwald, German Ott, Elías Campo, Pedro Jares

**Affiliations:** 1 Hematopathology Section, Department of Anatomic Pathology, Hospital Clínic, Institut d'Investigacions Biomèdiques August Pi i Sunyer, University of Barcelona, Barcelona, Spain; 2 Genomics Unit, Institut d'Investigacions Biomèdiques August Pi i Sunyer, University of Barcelona, Barcelona, Spain; 3 Department of Hematology, Hospital Clinic, University of Barcelona, Barcelona, Spain; 4 Institute of Pathology, University of Würzburg, Würzburg, Germany; 5 Department of Clinical Pathology, Robert-Bosch-Krankenhaus, and Dr. Margarete Fischer-Bosch Institute of Clinical Pharmacology, Stuttgart, Germany; Clinica Universidad de Navarra, Spain

## Abstract

**Background:**

Mantle cell lymphoma (MCL) is genetically characterized by the t(11;14)(q13;q32) translocation and a high number of secondary chromosomal alterations. The contribution of DNA methylation to MCL lymphomagenesis is not well known. We sought to identify epigenetically silenced genes in these tumours that might have clinical relevance.

**Methodology/Principal Findings:**

To identify potential methylated genes in MCL we initially investigated seven MCL cell lines treated with epigenetic drugs and gene expression microarray profiling. The methylation status of selected candidate genes was validated by a quantitative assay and subsequently analyzed in a series of primary MCL (n = 38). After pharmacological reversion we identified 252 potentially methylated genes. The methylation analysis of a subset of these genes (n = 25) in the MCL cell lines and normal B lymphocytes confirmed that 80% of them were methylated in the cell lines but not in normal lymphocytes. The subsequent analysis in primary MCL identified five genes (*SOX9*, *HOXA9*, *AHR*, *NR2F2*, and *ROBO1*) frequently methylated in these tumours. The gene methylation events tended to occur in the same primary neoplasms and correlated with higher proliferation, increased number of chromosomal abnormalities, and shorter survival of the patients.

**Conclusions:**

We have identified a set of genes whose methylation degree and gene expression levels correlate with aggressive clinicopathological features of MCL. Our findings also suggest that a subset of MCL might show a CpG island methylator phenotype (CIMP) that may influence the behaviour of the tumours.

## Introduction

Mantle cell lymphoma (MCL) is a well-defined lymphoid neoplasm characterized by a proliferation of mature B lymphocytes carrying the t(11;14)(q13;q32) translocation that leads to the overexpression of cyclin D1 [1]. In addition to this initial oncogenic event, MCL may carry a high number of secondary chromosomal and molecular alterations that influence the aggressive behaviour of this tumour [2]. Epigenetic marks, like DNA methylation and histone modifications, contribute to physiological and pathological states, including cancer [3]. In tumour cells, aberrant hypermethylation of stretches of CG-rich DNA, called CpG islands, located in promoter regions might result in inappropriate transcriptional silencing of tumour suppressor genes (TSG) [4]. Several studies, including genome-wide screening, have addressed the potential inactivation of specific TSG by methylation in MCL [5]–[7]. However, the limited number of cases or genes investigated in these studies does not allow to adequately determine the pathogenetic and clinical role of epigenetic gene silencing in this tumour.

Different genome-wide strategies, including different DNA microarrays formats and more recently bisulfite based massive parallel sequencing [8], [9], have been developed to identify genes silenced by CpG hypermethylation in human neoplasias. One of the first described approaches involves the pharmacological reversion of CpG methylation, accomplished by inhibition of DNA methyltransferase (DNMT) with drugs such as 5-aza-2′-deoxycytidine (5-aza-dC), coupled with the use of gene expression microarrays to identify methylated silenced genes that would be reactivated by drug treatment [10]. This reactivation seems to be reinforced by the concomitant treatment with histone deacetylase inhibitors (HDACi) like trichostatin A (TSA) [11]. This screening procedure has shown to be a powerful tool for the identification of TSG methylated in human cancers [12], [13].

In the current study, we sought to identify epigenetically silenced genes in MCL using an initial genome wide screening based on pharmacological reversion of CpG methylation and gene expression microarray analysis in MCL cell lines followed by the analysis of selected methylated genes in primary MCL and normal B lymphocytes. This approach has allowed us to identify a set of genes whose methylation degree and gene expression levels correlated with aggressive clinicopathologic features of the tumours and the outcome of the patients.

## Results

### Identification of potentially methylated genes in MCL cell lines by pharmacological reversion

To identify potentially methylated genes we focused on probe sets called “absent” in mock-treated cells but called “present” after 5-aza-dC and 5-aza-dC plus TSA. A higher reactivation of these probe sets was observed with the combination of 5-aza-dC and TSA (Figure S1). Finally, we selected 618 probe sets that were induced more than eight times in at least one MCL cell line compared to mock-treated cells (SignalLogRatio> = 3) (Figure S2). Twenty nine percent of these probe sets (n = 180) were not further considered due to poor annotation. Fourteen percent (n = 88) and 57% percent (n = 350) were mapped to sex and autosomal chromosomes, respectively (see Figure S2 for a description of the process). The probe sets mapped to sex chromosomes mainly interrogated cancer/testis antigens (CTAs) (Table S1).

We focused on the probe sets mapped to autosomal chromosomes that interrogated a total of 331 genes (Table S2 and Table S3). A canonical CpG island around the transcription start site was found in 252 (76%) of these genes (Table S2). We have calculated that only 53% protein consensus coding sequence (CCDS) genes interrogated by the HU133plus 2.0 would contain **a CpG island** around the transcription start site [14]. This result would confirm that we obtained a significant enrichment for CpG islands containing genes (*P*<1e-10). A pathway analysis identified that the top molecular and cellular functions represented by our candidate genes were cell death, cell cycle, and cellular growth and proliferation.

### CpG promoter analysis of candidate genes in cell lines and primary MCL

To determine the presence of promoter methylation in genes induced following drug treatment we analyzed 25 candidate genes containing CpG islands using a MassArray assay. This set was selected following a bibliography search and included genes described as regulated by hypermethylation in human tumours, and genes whose function might suggest a putative role as TSG. In total 46 amplicons were designed to interrogate 25 genes in seven MCL cell lines and in a pooled DNA sample derived from purified CD19+ B lymphocytes obtained from four different tonsils.

**Figure 1:**
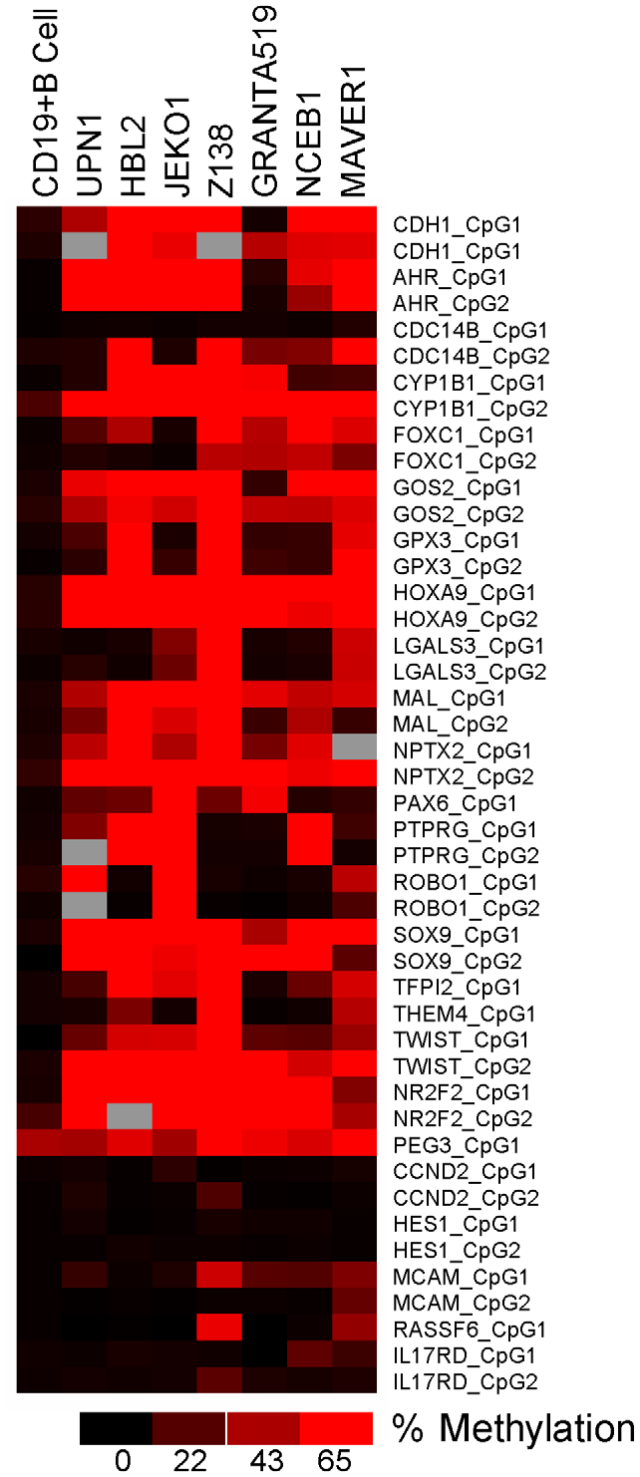
Heat map showing the percentage of methylation for the 45 amplicons analyzed. Amplicons in red showed a methylation degree higher than 65%.

Forty-five of the 46 amplicons were successfully analyzed. The majority of CpG of all amplicons were unmethylated in normal DNA, but showed different methylation patterns in MCL cell lines (Figure 1). A CpG island was considered as methylated when at least one amplicon showed a median methylation percentage of all CpG units equal or higher than 65%. Twenty genes out of 25 (80%) showed hypermethylation in at least one MCL cell line, whereas all of them (*CDH1*, *AHR*, *CDC14B*, *CYB1B1*, *FOXC1*, *G0S2*, *GPX3*, *HOXA9*, *LGALS3*, *MAL*, *NPTX2*, *PAX6*, *PTPRG1*, *ROBO1*, *SOX9*, *TFPI2*, *THEM4*, *TWIST1*, and *NR2F2*) but one (*PEG3*) were unmethylated in normal DNA. Only five genes did not show significant methylation in any of the seven MCL cell lines (*CCND2*, *HES1*, *MCAM*, *RASSF6* and *IL17R10*) supporting the good relationship between pharmacological reversion of CpG methylation and reactivation of gene expression in these MCL cell lines.

**Figure 2:**
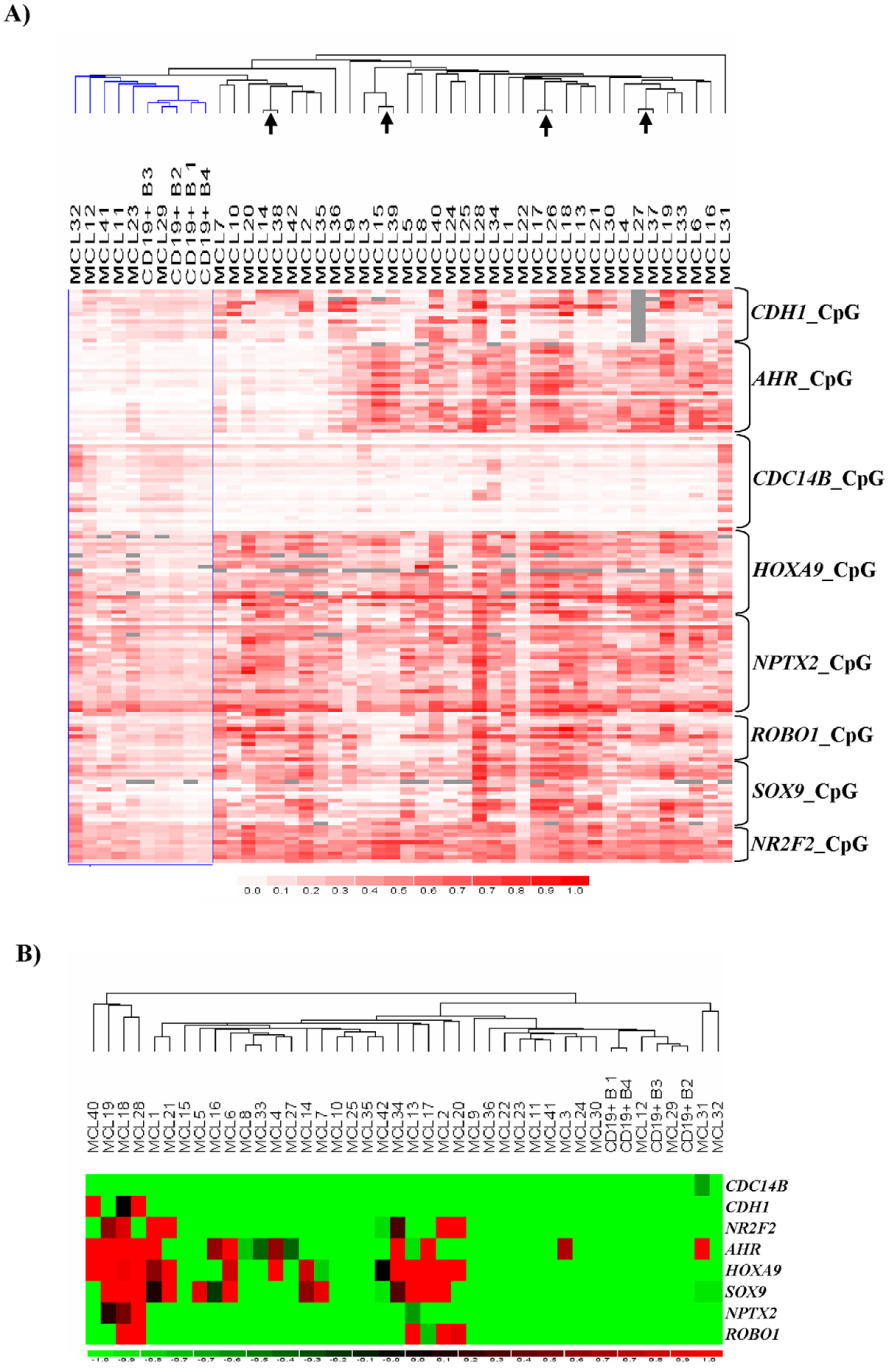
CpG methylation in primary MCL. A) Hierarchical clustering of MCL samples and normal CD19+ B cells. Row methylation values are represented in a heat map, unmethylation (white) and full methylation (red). Blue colour highlights the cluster containing the four normal samples. The black arrows indicate the four duplicate DNA samples. B) Values represent the summary of the CpG unit methylation data for each gene. The samples were clustered using the data standardized for the cut-off used to define gene methylation.

To validate the methylation events identified in the MCL cell lines we investigated the methylation status of eight genes (*CDH1*, *AHR*, *CDC14B*, *HOXA9*, *ROBO1*, *SOX9*, *NR2F2*, and *NPTX2*) in 38 primary MCL using one of the previously designed amplicons for each gene (Figure 2A) that showed specific hypermethylation in at least two MCL cell lines (Figure 1). We included four duplicate samples to test the reproducibility of the whole approach. The correlation analysis (r>0.928, *P*<0.001) and the close clusterization of duplicated samples (Figure 2A) confirmed the high reproducibility of the assay. The unsupervised analysis of the methylation profile showed two major clusters one of them contained all the normal samples and a small subset (n = 6) of primary tumours that appear to have a lower methylation profile than the rest of MCL (Figure 2A). The study also showed that 7 of the 8 genes showed different degrees of methylation in primary tumours.

### Gene methylation and clinicopathologic characteristics of MCL

We analyzed whether the methylation status of single genes correlated with clinicopathologic parameters of the tumours (Table 1). A gene was considered to be methylated in primary MCL when the methylation level detected was at least above 30% and three times higher than the methylation degree observed in normal CD19+ lymphocytes. Three genes appeared to be frequently methylated in primary MCL. *SOX9* was found methylated in 13 out of 38 (35%), *HOXA9* in 15 out of 38 (41%), and *AHR* in 12 out of 38 (32%) samples (Figure 2B). The other four genes showed methylation in a smaller subset of patients, *NR2F2* in 7 out of 38 (19%), *ROBO1* in 5 out of 38 (14%), *NPTX2* in 3 out of 38 (8%), and *CDH1* in 3 out of 36 (8%) samples. *CDC14B* was not found methylated in primary MCL.

**Table 1:** Relationship between gene methylation and clinicopathological features of MCL.

		*SOX9*		*HOXA9*		*AHR*		No Methylated Genes	
		unM	M	unM	M	unM	M	≤1	>1
Ki-67 (%)	n	17	12	17	12	19	10	17	12
	mean±sd	16±13	46±26*	16±13	46±26*	25±23	34±27	16±13	46±26*
CA†	n	13	11	13	11	16	8	13	11
	mean±sd	4.5±2.9	7.8±3.2*	4.2±2.6	8.2±3.0*	5.4±3.5	7.4±3.0	4.2±2.6	8.2±3.0*
OS‡	n	18	11	16	13	19	10	17	12
	mean	74.4	29.8	79.4	31.5	69.0	33.8	75.8	32.6
	(95% CI)§	49.7–99.2	9.9–49.7*	52.9–106	12.4–50.5*	44.2–93.9	19.5–48.2*	50–102	12–53*

† CA: Number of chromosome alterations,

‡ OS: Overall survival in months,

§ 95% CI Confidence interval. unM: unmethylated, M: methylated,

*statistically significant at p<0.05.

**Figure 3:**
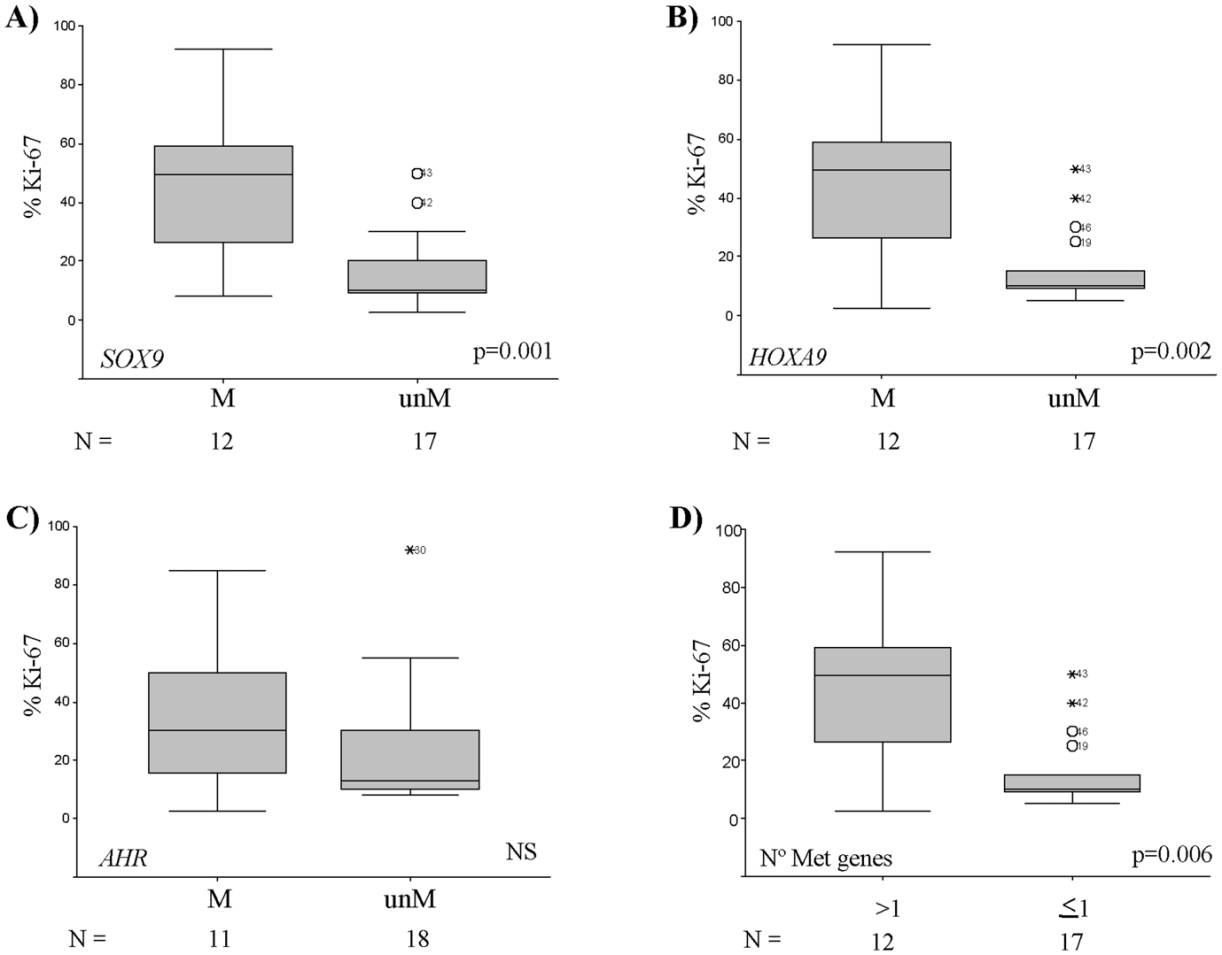
Box plots representing the median and range of the Ki67 index for the groups of primary MCL with methylated (M) and unmethylated (unM) genes. A) *SOX9*, B) HOXA9, and C) *AHR*. D) Proliferation index of MCL subgroups according to the number of concomitant methylated genes (≤1 M: none or only one methylated gene; >1 M: more than one methylated gene).

MCL with methylated *SOX9* (p = 0.001) or *HOXA9* (*P* = 0.002) showed significant higher proliferation (Ki-67 index) than unmethylated tumours (Figure 3 and Table 1). Furthermore, the number of Ki-67 positive cells correlated significantly with the methylation levels of *SOX9* (r_s_ = 0.497, *P* = 0.006), *HOXA9* (r_s_ = 0.496, *P* = 0.01), *NPTX2* (r_s_ = 0.581, *P* = 0.001), *NR2F2* (r_s_ = 0.460, *P* = 0.012), and *ROBO1* (r_s_ = 0.388, *P* = 0.038).

In a series of 24 MCL with available CGH data, tumours with methylated *SOX9* (*P* = 0.023) or *HOXA9* (*P* = 0.006) showed a higher number of chromosomal abnormalities than unmethylated cases (Table 1). Concordantly to the above described associations, the four blastoid cases showed higher degree of methylation of *SOX9* (*P* = 0.028), *HOXA9* (p = 0.05), and *ROBO1* (*P* = 0.037) than classical MCL.

**Figure 4:**
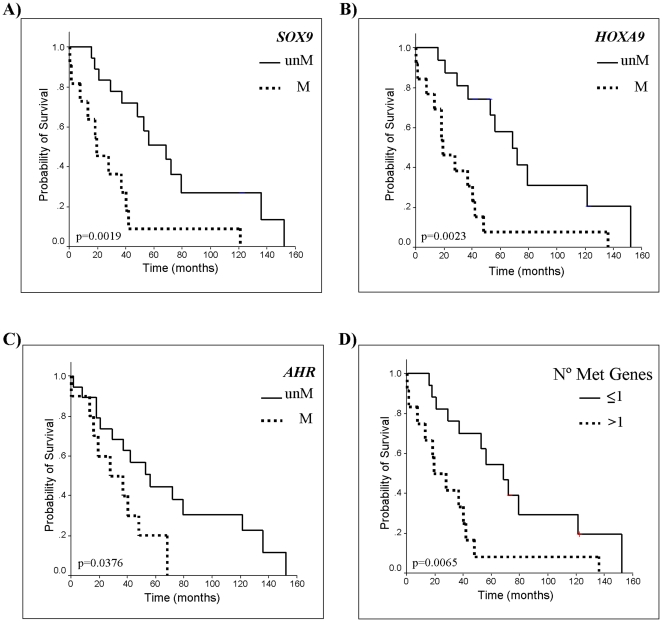
Kaplan-Meier survival curves according to gene methylation status of A) *SOX9*, B) *HOXA9*, and C) *AHR* (M methylated, unM unmethylated). D) Overall survival (OS) according to the number of concomitant methylated genes (≤1 M: none or only one methylated gene; >1 M: more than one methylated gene).

Survival information was available in 29 primary MCL (Figure 4). MCL with methylation of *SOX9* (*P* = 0.0019), *HOXA9* (*P* = 0.0023), or *AHR* (*P* = 0.0376) had a significantly shorter OS compared to patients with unmethylated genes (Figure 4). Although the methylation status of three out of the four remaining genes (*NR2F2*, *NPTX2*, and *CDH1*) also showed a tendency to be associated with shorter OS, it did not reach statistical significance.

The methylation status of *SOX9*, *HOXA9*, and *AHR* were compared with the Ki-67 index in bivariate COX regression analyses. *SOX9* methylation, but not Ki-67, remained as a significant independent survival predictor (Relative Risk (RR) = 3.84; *P* = 0.0086)). When *HOXA9* or *AHR* methylation were compared with the Ki-67 index, both genes and Ki-67 retained their independent prognostic value (*HOXA9*, RR = 3.48; *P* = 0.0129; Ki-67, RR = 2.9, *P* = 0.036) (*AHR*, RR = 3.72; p = 0.0164; Ki-67 RR = 3.85; *P* = 0.0075).

### CpG methylation profile and clinicopathologic parameters in MCL

**Figure 5:**
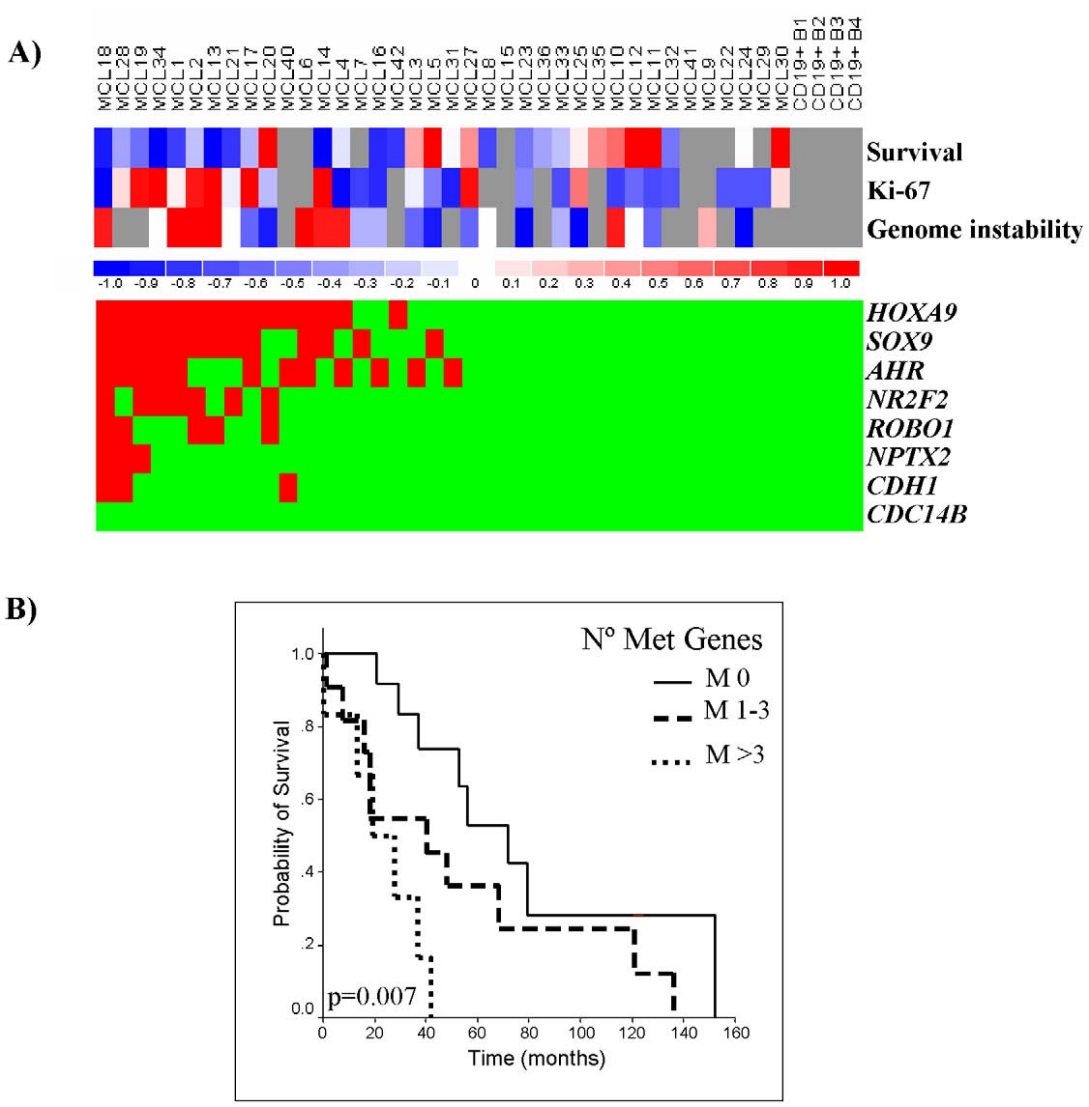
Characterization of MCL according to the methylation status of analyzed genes and their respective clinicopathological features. A) Distribution of the number of methylated genes in primary MCL. The presence of methylation is represented in red and the absence in green. Clinicopathological variables are represented in a blue-red heat map. B) Kaplan-Meier survival curve of MCL patients according to the accumulation of methylated genes (Met genes): M0 no methylated genes, M1-3 one to three methylated genes, M>3 more than 3 methylated genes.

We observed that gene methylation events tended to occur in the same primary MCL (Figure 5A). To determine whether the number of methylated genes had an impact on the clinicopathological features of the patients, we classified the tumours into two groups. One subset included cases with none or only one methylated gene (n = 24) whereas the second group consisted of cases with two or more methylated genes (n = 14). MCL that accumulated methylated genes had higher proliferation (*P* = 0.002), increased number of chromosomal abnormalities (*P* = 0.006) and the patients had shorter overall survival (OS) than cases with one or none methylated genes (*P* = 0.0065) (Figure 3D). In a multivariate COX regression analysis, both the presence of methylation in more than one gene and Ki-67 index retained their predictive value for OS (RR = 3.48, *P* = 0.0129; RR = 2.9, *P* = 0.0036). To further explore the relationship between the accumulation of methylation events and survival we divided the cases in three groups corresponding to cases with no methylated genes (n = 18), cases with methylation of 1 to 3 genes (n = 14), and cases with more than 3 methylated genes (n = 6). The accumulation of methylated genes was significantly associated with a shorter OS of the patients (*P* = 0.007) (Figure 5B).

### Gene expression of methylated genes, association with promoter methylation and survival

To explore the relationship between promoter methylation and gene expression in primary tumours we performed a qRT-PCR analysis of *SOX9*, *HOXA9*, *AHR*, *ROBO1*, and *NR2F2* in a series of 36 MCL. We found a modest but statistically significant inverse correlation between methylation and mRNA levels for *SOX9* (r_s_ = −0.431, *P* = 0.02), *AHR* (r_s_ = −0.411, *P* = 0.027), and *NR2F2* (r_s_ = −0.387, *P* = 0.038). The inverse correlation observed for *ROBO1* when all CpG units were considered was not significant. However, when single CpG units were analyzed, the methylation of one of the twelve *ROBO1* CpG units present in the amplicon showed a strong inverse correlation with gene expression (r_s_ = −0.575, *P* = 0.001). Neither global nor single CpG unit methylation of *HOXA9* correlated with gene expression. Normal lymph node samples showed significant higher expression levels of SOX9, AHR, NR2F2, HOXA9, and ROBO1 than primary methylated MCL (Figure S3).

Next, we investigated whether the mRNA expression levels of the methylated genes would correlate with the OS of the patients. Patients were divided in low and high groups according to the median expression of the genes in the tumours. Low mRNA levels of SOX9 or ROBO1 were associated with a shorter survival of the patients (*P* = 0.0015 and *P* = 0.0485, respectively). Low levels of NR2F2 and AHR showed a similar tendency but did not reach statistical significance.

## Discussion

In the present study, we have designed a comprehensive strategy to uncover novel TSG methylated in MCL. The initial pharmacological unmasking of methylated genes in cell lines was followed by a validation study in primary tumours, allowing us to identify a set of genes frequently methylated in MCL. The methylation and gene expression levels of these genes correlated with aggressive clinicopathological variables of the tumours. Moreover, our results suggest for the first time that a subset of MCL might show a CpG island methylator phenotype associated with higher proliferation, more complex karyotypes and poor clinical outcome.

The low frequency or absence of methylation in critical TSG genes commonly inactivated by mutation or deletion in MCL has challenged the role that DNA methylation might play in MCL lymphomagenesis [2]. Although several studies have analyzed the methylation status of few genes in MCL no association with clinicopathologic features have been described [5], [6]. Recently two groups have reported the use of genome-wide approaches to study DNA methylation in hematological neoplasms including a small series of MCL [15], [16]. The authors concluded that germinal center lymphomas display a significantly higher methylation degree than pre-germinal center tumours such as MCL. However, the relevance of DNA methylation in MCL remains to be clarified.

The combination of CpG methylation reversion by 5-aza-dC with microarray analysis has been successfully applied in different tumour cell lines to identify methylated genes [17], [18]. We performed this approach in a set of seven MCL cell lines, but in addition to the treatment with 5-aza-dC, we treated the cells with the combination of 5-aza-dC and TSA. Our initial microarray data analysis indicates that the previously described synergy between 5-aza-dC and TSA also seems to occur in MCL cell lines [11], [12]. Our approach is more comprehensive than a previously published work where the methylation unmasking was performed in a single MCL cell line using only 5-aza-dC [19].

Our screening performance was supported by two of our initial results. First, the majority of the selected probe sets mapping to sex chromosomes corresponded to CTAs. These genes are commonly expressed in testis and/or placenta, but infrequently expressed in non-germline normal tissues [20], and their expression is reactivated by 5-aza-dC [21]. The second evidence was that a significant proportion of the candidate genes located in autosomal chromosomes contained a canonical 5′ CpG island (76%), whereas we estimated that only 53% of CCDS genes interrogated in the HU133 plus 2.0 microarray would contain one. In addition, the identification of DNA methylation in a high proportion of the genes analyzed in MCL cell lines (80%) strongly supports the effectiveness of our approach. Gene methylation events may occur during the establishment of cancer cell lines, but in some models there is a good relationship between the methylation events detected in cell lines and the methylation pattern of the primary tumours from which the cell lines were derived [22]. We observed a good correlation between methylation events in cell lines and primary MCL since we found partial methylation in 80% of the genes analyzed in primary MCL. This association suggests that MCL cell lines are a good model to study epigenetic aspects of MCL. All these results confirmed that our approach is robust and reliable, and suggests that we may be able to identify additional epigenetically regulated genes in the initial set of 252 methylated candidates. In addition, the pathway analysis showed that the top molecular and cellular functions overrepresented in our candidate gene list were cell death, cell cycle and cellular growth and proliferation. Recently, Leschenko et al. have reported the global methylation of a series of primary MCL using the HELP assay [23]. The authors described that the phenomenon of gene hypomethylation was more frequent than the hypermethylation in primary MCL. Furthermore, they identified a group of genes that were hypermethylated in primary MCL compared with naïve B-cells. Our approach, based in the pharmacological reversion of DNA methylation, only allow us to identify hypermethylated genes precluding the evaluation of hypomethylation in our series. When we compared our list of potentially hypermethylated genes with the genes described as hypermethylated in MCL by Leschenko we observed that 28 genes of our study were also described as methylated in MCL by Leschenko et al.

The five genes that we found methylated in more than 10% of primary MCL had been reported to be methylated in different tumour models, but none of them were previously analyzed in MCL. *SOX9* methylation has been described in bladder tumours and in FL [24], [25]. Moreover, *SOX9* has shown a tumorigenicity inhibitor effect in different tumour cells, suggesting a potential tumour suppressor role [26], [27]. Similarly, high levels of *AHR* promoter methylation have been described in acute lymphoblastic leukemia (ALL) and chronic myeloid leukemia (CML) cell lines, as well as in primary ALL [28]. In solid tumours several evidences suggest that *HOX9A* behaves like a TSG. In that sense, *HOXA9* hypermethylation has been described in ovarian tumours and in squamous cell lung carcinomas [29], [30]. Interestingly, *HOXA9* methylation has also been reported in FL and AML [25], [31]. Several evidences, including the detection of *ROBO1* methylation in primary solid tumours and the predisposition of mice carrying an inactivated *ROBO1* allele to generate lymphomas, support the role of this gene as a classical TSG [32]–[34]. *NR2F2* hypermethylation has recently been described in breast carcinomas and AML [31], [35]. Now, we report a significant association between the methylation status of these genes and different clinicopathological features of primary MCL, together with an inversed correlation between gene expression and methylation levels, suggesting that this methylation might play a role in the pathogenesis of MCL. In addition, the determination of DNA methylation in these genes might be of prognostic value in MCL, but studies in large series of primary cases are required.

The methylation pattern analysis showed that gene methylation does not occur stochastically and concordant methylation events seem to take place in the same primary MCL. The accumulation of methylated genes was associated with high levels of proliferation, increased number of chromosomal abnormalities, and lower OS. This scenario is reminiscent of the condition defined in epithelial tumours as CpG island methylator phenotype that is characterized by the accumulation of methylated genes associated with worse prognosis [36]. Since all samples were selected for the DNA extraction based on the histological observation of high tumor cell content, the identification of primary cases without or with low methylation can not be due to a reduced number of tumor cells. This phenotype has not only been described in solid tumours but also in hematological neoplasms such AML and ALL. Our results would suggest that it might also occur in lymphomas, and specifically in MCL. Subsequent studies in larger series of independent cases are required to confirm these findings.

In summary, we have identified new methylated genes in primary MCL that are associated with several aggressive clinicopathological variables. The identification of new methylated genes associated with the clinical behaviour in MCL patients suggests that epigenetic changes may play an important role in the pathogenesis of these tumours and may be the target of new therapies. Moreover, we present evidence suggesting that some MCL cases might display a CpG island methylator phenotype -like phenotype associated with poor clinical outcome.

## Materials and Methods

### Cell lines and case selection

Seven well characterized MCL cell lines (UPN1, JEKO1, HBL2, GRANTA519, MAVER1, NCEB, and Z138) were used for the initial genome wide screening [37]. Tumour tissue specimens from 38 MCL patients were obtained from the Tumour Bank of the Department of Pathology of the Hospital Clínic of Barcelona, and the Institute of Pathology, University of Würzburg. The minimum percentage of tumor cells that were present in the biopsy sections used for DNA extraction were evaluated following microscopic observation and in all cases this percentage was higher than 80%. Normal reactive tonsils from adult healthy individuals were used as a source of normal B-cells. Briefly, mononuclear fraction was isolated using Ficoll from the solution obtained by washing the tonsil nodules with RPMI1640 (BioWhittaker, Cambrex) supplemented with 16.5 mM HEPES (Boehringer Mannheim, Germany). Normal CD19+ B lymphocytes were obtained by magnetic cell sorting from four tonsil samples using CD19 human microbeads and autoMACS separator (MACS, Miltenyi Biotec) following manufacturer's protocol. The characterization of these tumours, including Ki-67 index and comparative genomic hybridization (CGH) data had been described previously [38]. The study was approved by institutional review boards at the Hospital Clinic of Barcelona and the Institute of Pathology of the University of Würzburg. Written informed consent was obtained from all participants and the ethics committees approved this consent procedure in accordance with the principles of the Declaration of Helsinki.

### Pharmacological reversion of CpG methylation

The pharmacological reversion of the potentially methylated genes in the cell lines was performed using 5-aza-dC (100 nM for 72 h, Sigma, Steinheim, Germany) and 5-aza-dC (100 nM for 72 h) followed by incubation with TSA (300 nM the last 24 h, Sigma). Due to the poor chemical stability of 5-aza-dC, fresh medium containing 5-aza-dC was added every 24 hours. These drug treatment conditions were established after a preliminary study of *XIST* gene expression in MCL cell lines derived from males by quantitative reverse-transcription polymerase chain reaction (qRT-PCR) (see Supporting Information S1 and Figure S4). Mock-treated cells were used as reference. Total RNA was processed and hybridized onto HG-U133 Plus 2.0 microarrays following the manufacturer's recommendations (Affymetrix Inc., Santa Clara, Ca). All array data is MIAME compliant and raw data had been deposited in GEO database (GSE22038).

### Promoter methylation analysis

Promoter methylation analysis of twenty five candidate genes was performed with the MassArray® EpiTYPER® assay (Sequenom, San Diego, CA). Amplicons were designed using the EpiDesigner application (www.epidesigner.com). Two amplicons were designed for 19 genes, one amplicon for five genes, and three amplicons for one gene. The amplicons analyzed are described in Table S4. Each analyzed amplicon contains several CpGs and the EpiTyper software indicates the percentage of methylation for each CpG unit. The degree of methylation of each gene was defined as the median methylation percentage of the whole set of CpG units in the amplicons.

### Study of gene expression by qRT-PCR

Total RNA isolation and quality control analysis with the Bioanalyzer 2100 (Agilent, Palo Alto, CA) were performed as described before [38]. mRNA expression was analyzed by qRT-PCR using designed human Taqman® Gene expression Assays (Applied Biosystems, Foster City, CA).

### Bioinformatic and statistical analysis

The microarray data analysis was performed with different applications including GeneChip Operating Software (GCOS, Affymetrix®) and DNA-Chip Analyzer (dChip) (see Supporting Information S1). An algorithm based on Signal and Detection Call was applied to select the candidate probe sets. Probe sets that passed our filter criteria were included independently that other probe sets of the same gene could be filtered out. The Genome Browser and Galaxy platforms were used to identify the candidate genes and the proportion of consensus coding sequence (CCDS) genes that contain CpG islands around 2 Kb of the transcription start site and which are interrogated in the HU133plus2.0 array [39], [14]. Pahtway analysis was performed with the Ingenuity software. The statistical evaluation was performed using nonparametric tests. The means were compared using Mann-Whitney test. Fisher's exact test was used for comparison between categorical data. Overall survival (OS) was estimated using the Kaplan-Meier method and compared by means of the log-rank test. Cox proportional-hazards model was used to analyze prognostic factors. The relationship between two variables was computed using Pearson correlation (r) and nonparametric Spearman's rho correlation (r_s_). The level of significance was set at 0.05, and all calculations were performed with the SPSS software package (version 11; SPSS Inx, IL).

## Supporting Information

Table S1 **Selected Probes Sets that mapped to sexual crhomosomes.**
(XLS)

Table S2 **Candidate genes containing CpG islands localized to autosomal chromosomes. In bold and underlined genes validated in MCL cell lines.**
(XLS)

Table S3 **Non CpG islands genes.**
(XLS)

Table S4 **Amplicons analyzed in 25 genes indicating the size of the amplicons (Length) and the number of CpG locis (CpGs).** In bold are indicated the amplicons analyzed in primary MCL
(XLS)

Figure S1 **A potential synergic effect between 5-aza-dC and TSA.** Reactivation levels of probe sets call absent in mock treated cells that turn to be called present after both drug treatments. Red color means higher levels than green color comparing the gene expression levels in both treatment conditions. N means number of probe sets.
(TIF)

Figure S2 **Flowchart describing the steps followed to select the final eight genes analyzed in primary MCL.**
(TIF)

Figure S3 **Box plots representing the median and range of relative gene expression [A)** ***SOX9*** **; B)** ***AHR*** **; C)** ***NR2F2*** **; D)** ***ROBO1*** **; E)** ***HOXA9*** **] for the groups of primary MCL gene methylation status (M: methylated and umM: unmethylated), and normal lymph nodes (LN).**
(TIF)

Figure S4 ***XIST*** **mRNA expression following 5-aza-dC titration.** a) qRT-PCR of *XIST* mRNA was performed after 5-aza-dC titration (30 nM, 60 nM, and 100 nM), and XIST mRNA levels were compared to mock treated cells. b) Heat Map showing XIST mRNA levels detected in HBL2 by 6 probe sets after mock, 60 nM 5-aza-dC, 100 nM 5-aza-dC, and 100 nM 5-aza-dC+TSA treatment. The microarray data (mean of the six probes sets) comparing drug versus mock treatment is represented in a bar plot.
(TIF)

Supporting Information S1 **Identification of experimental conditions for pharmacological reversion of gene expression in MCL cell lines.**
(DOC)
