# Supplementary figures and images for: Identification of Methylated Genes Associated with Aggressive Clinicopathological Features in Mantle Cell Lymphoma
Source: PLoS One. 2011 May 16;6(5):e19736. doi: 10.1371/journal.pone.0019736 (PMC3095614; doi:10.1371/journal.pone.0019736)

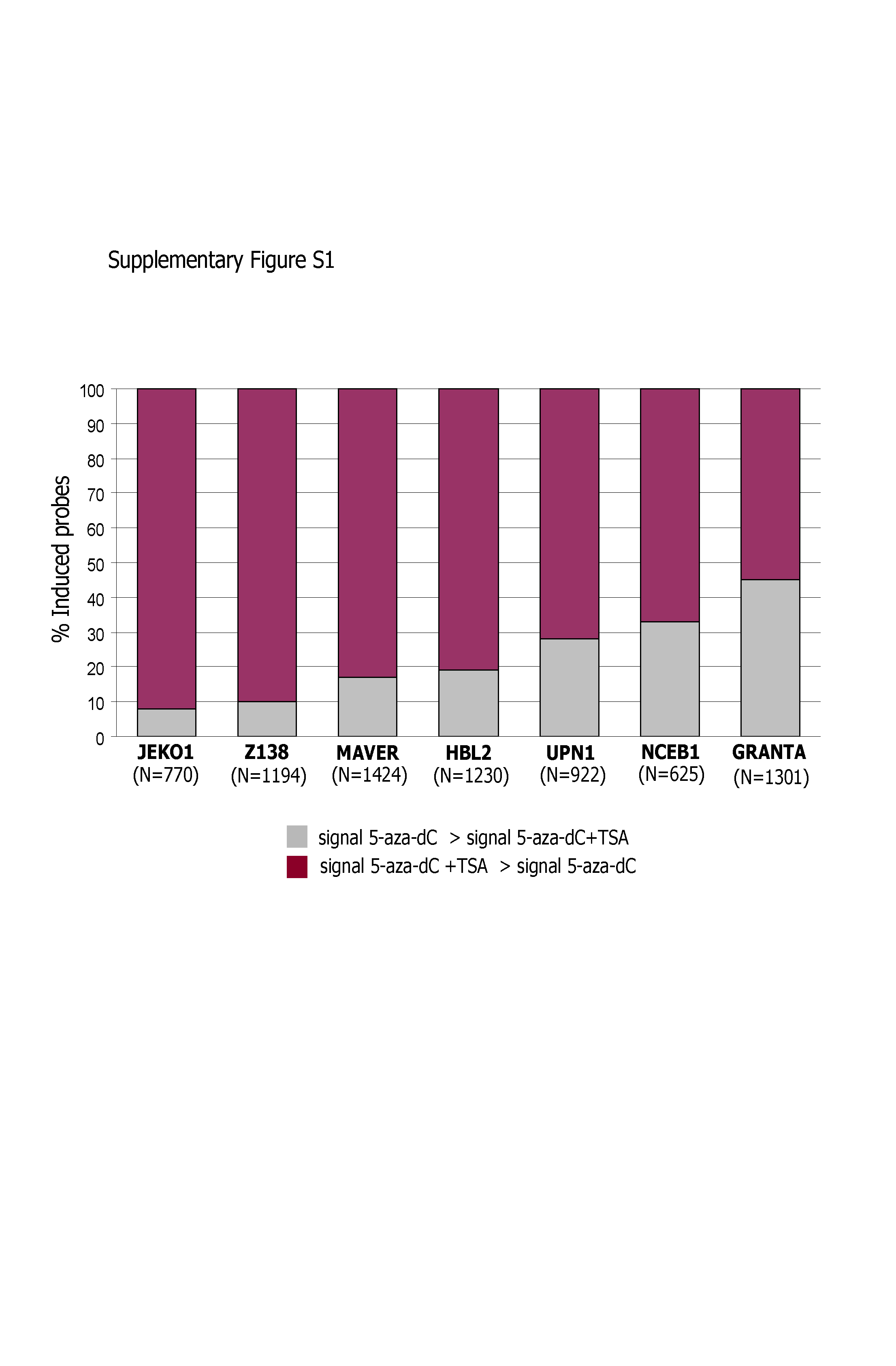

Supplement: Figure S1 — A potential synergic effect between 5-aza-dC and TSA. Reactivation levels of probe sets call absent in mock treated cells that turn to be called present after both drug treatments. Red color means higher levels than green color comparing the gene expression levels in both treatment conditions. N means number of probe sets. (TIF) [file pone.0019736.s005.tif]

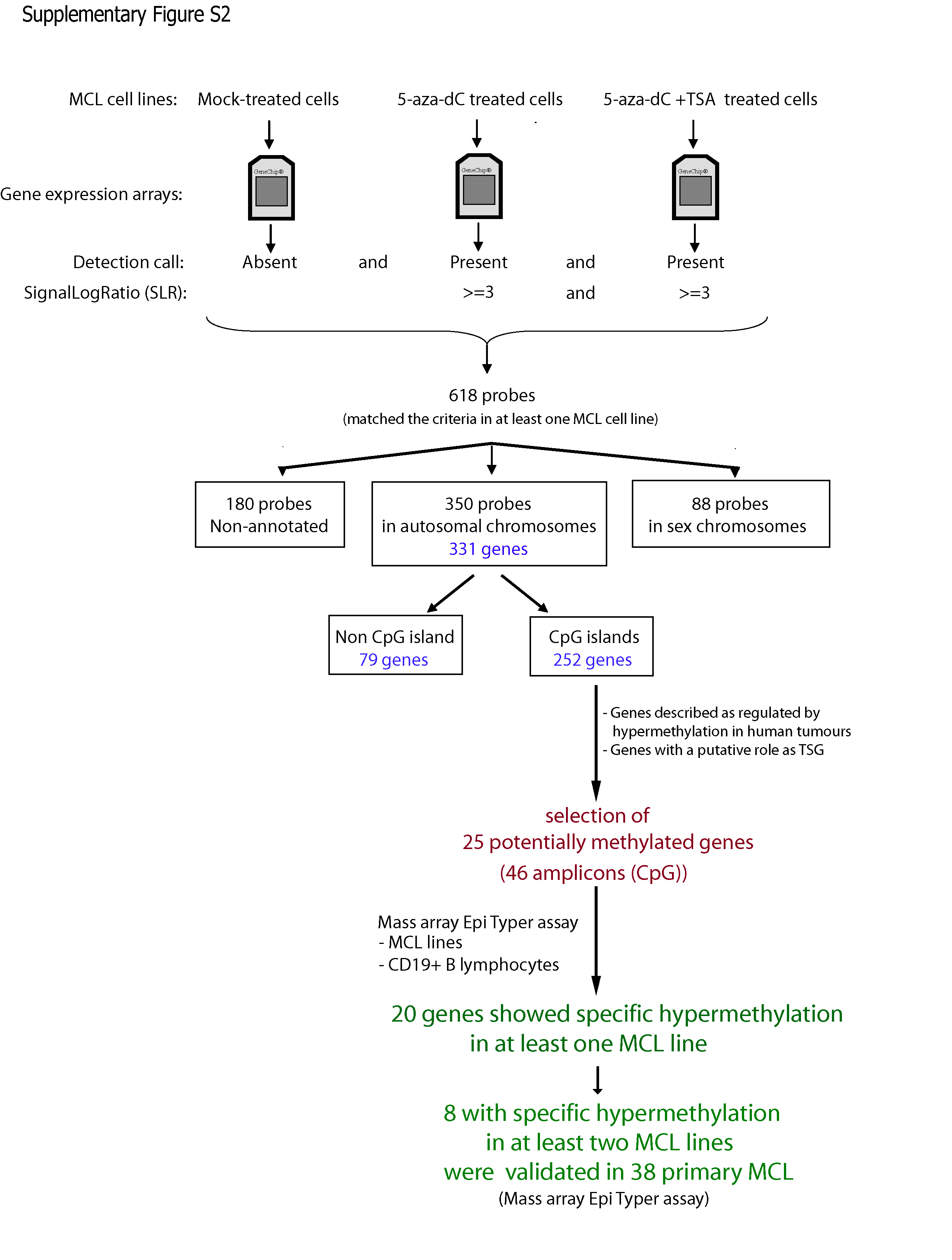

Supplement: Figure S2 — Flowchart describing the steps followed to select the final eight genes analyzed in primary MCL. (TIF) [file pone.0019736.s006.tif]

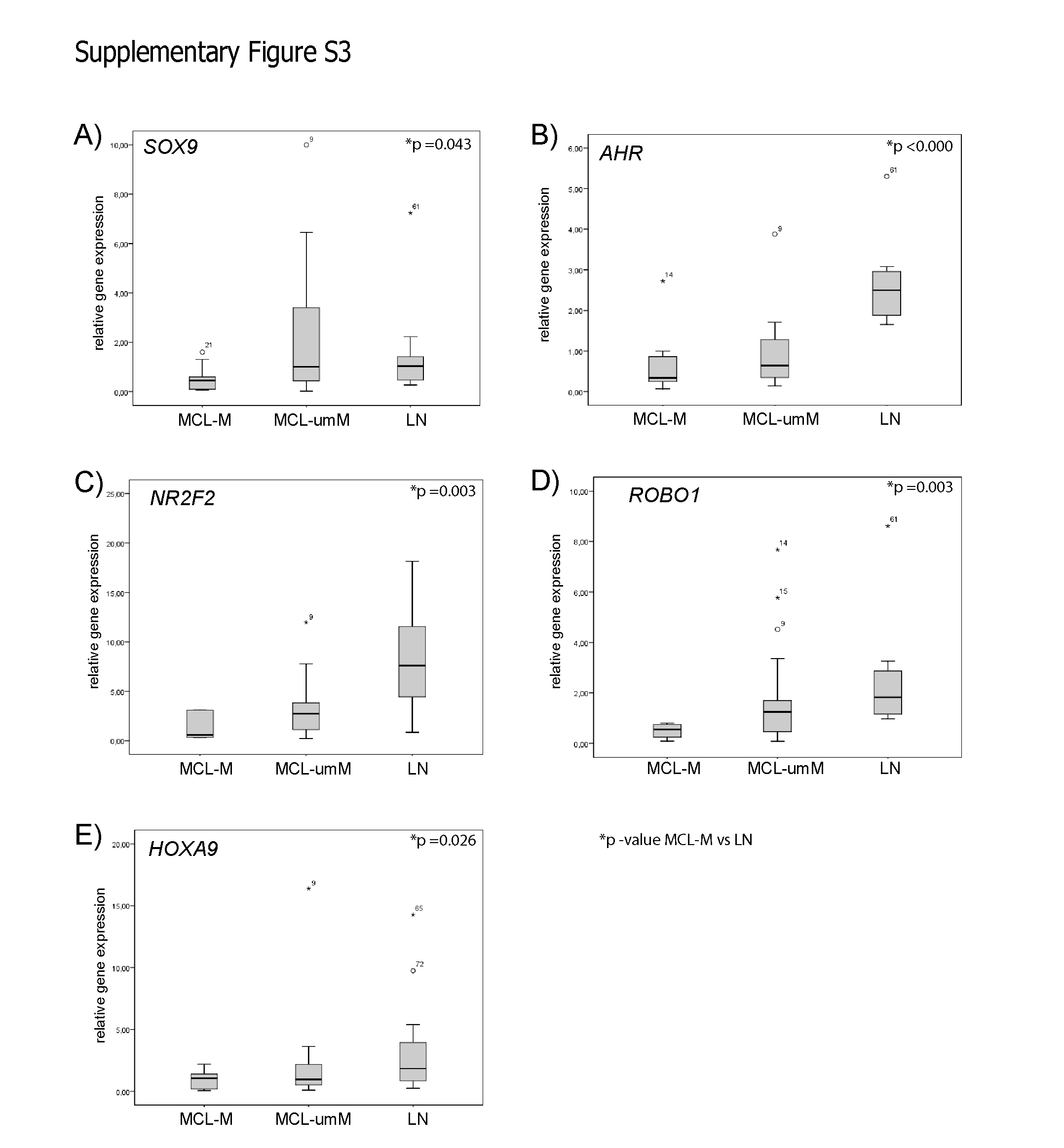

Supplement: Figure S3 — Box plots representing the median and range of relative gene expression [A) SOX9 ; B) AHR ; C) NR2F2 ; D) ROBO1 ; E) HOXA9 ] for the groups of primary MCL gene methylation status (M: methylated and umM: unmethylated), and normal lymph nodes (LN). (TIF) [file pone.0019736.s007.tif]

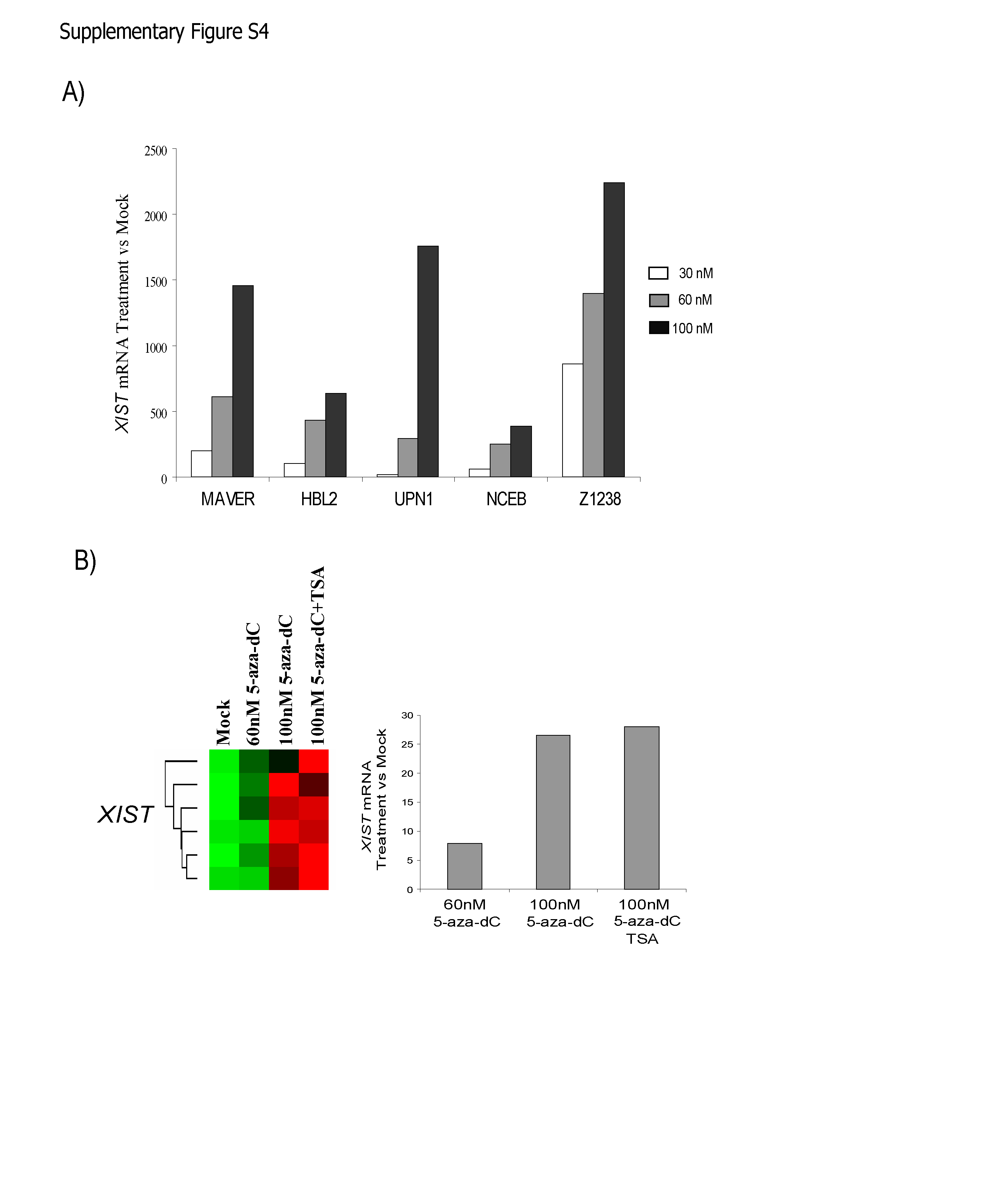

Supplement: Figure S4 — XIST mRNA expression following 5-aza-dC titration. a) qRT-PCR of XIST mRNA was performed after 5-aza-dC titration (30 nM, 60 nM, and 100 nM), and XIST mRNA levels were compared to mock treated cells. b) Heat Map showing XIST mRNA levels detected in HBL2 by 6 probe sets after mock, 60 nM 5-aza-dC, 100 nM 5-aza-dC, and 100 nM 5-aza-dC+TSA treatment. The microarray data (mean of the six probes sets) comparing drug versus mock treatment is represented in a bar plot. (TIF) [file pone.0019736.s008.tif]
