# Supplementary material for: Identification of Methylated Genes Associated with Aggressive Clinicopathological Features in Mantle Cell Lymphoma
Source: PLoS One. 2011 May 16;6(5):e19736. doi: 10.1371/journal.pone.0019736 (PMC3095614; doi:10.1371/journal.pone.0019736)
Supplement: Supporting Information S1 — Identification of experimental conditions for pharmacological reversion of gene expression in MCL cell lines. (DOC) [file pone.0019736.s009.doc]

**SUPPLEMENTARY MATERIAL**

**RESULTS**

**Identification of experimental conditions for pharmacological reversion of gene expression in MCL cell lines.**

First of all, it was necessary to identify the concentration of 5-aza-dC with a higher effect in re-expression of epigenetically silenced genes that allowed reasonable cell viability (viability ≥50%). Cell viability was measured by double annexin-V and propidium iodide staining by flow citometry using Annexin V FITC Kit (Bender Medsystems®, Vienna, Austria) following manufacturer instructions. We performed a titration experiment with 30nM, 60nM, and 100nM of 5-aza-dC. The expression of *XIST*, which is involved in the silencing of X chromosome in female cells, was used as control to follow the epigenetic silencing reversion produced by drug treatment. Since *XIST* is epigenetically silenced in male cells, we performed the experiment in five MCL cell lines derived from males (HBL2, UPN1, Z138, NCEB and MAVER). We studied the mRNA levels of *XIST* after drug treatment by qRT-PCR and observed that the induction of *XIST* occurs clearly at 60nM and 100nM (Supplementary Figure S4A). The effect on cell viability was less pronounced at 60nM (data not shown). It has been demonstrated that the dynamic range of qRT-PCR is not the same as microarrays.(1) For this reason, a pilot microarray hybridization experiment was performed using HBL2 at 60nM and 100nM. As it is shown in Supplementary Figure S4B the induction was more clearly detected in microarrays when a concentration of 100nM 5-aza-dC was used. In consequence, the seven MCL cell lines (UPN-1, JEKO1, HBL2, GRANTA519, MAVER1, NCEB1, and Z138) were treated with 100nM 5-aza-dC during 72 hours, and mRNA extracted from untreated and treated samples was hybridized onto microarrays. This drug concentration is lower than the already described by other authors in different tumors.(2,3) It was reported previously that the combination of 5-aza-dC treatment with TSA could show a synergic effect in the reactivation of genes repressed by hypermethylation. For this reason, a third array was hybridized with mRNA from cells treated with 100 nM 5-aza-dC followed with a 300 nM TSA treatment for another 24 hours.

**Supplementary Figure Legends**

**Supplementary Figure S1. A potential synergic effect between 5-aza-dC and TSA.** Reactivation levels of probe sets call absent in mock treated cells that turn to be called present after both drug treatments. Red color means higher levels than green color comparing the gene expression levels in both treatment conditions. N means number of probe sets.

**Supplementary Figure S2**. Flowchart describing the steps followed to select the final eight genes analyzed in primary MCL.

**Supplementary Figure S3. Box plots representing the median and range of relative gene expression** [A). *SOX9*; B) *AHR*; C) *NR2F2*; D) *ROBO1*; E) *HOXA9*] for the groups of primary MCL gene methylation status (M: methylated and umM: unmethylated), and normal lymph nodes (LN).

**Supplementary Figure S4. *XIST* mRNA expression following 5-aza-dC titration.**

a) qRT-PCR of *XIST* mRNA was performed after 5-aza-dC titration (30nM, 60nM, and 100nM), and XIST mRNA levels were compared to mock treated cells. b) Heat Map showing XIST mRNA levels detected in HBL2 by 6 probe sets after mock, 60nM 5-aza-dC, 100nM 5-aza-dC, and 100nM 5-aza-dC+TSA treatment. The microarray data (mean of the six probes sets) comparing drug versus mock treatment is represented in a bar plot.

Reference List

1. Wang Y, Barbacioru C, Hyland F et al. Large scale real-time PCR validation on gene expression measurements from two commercial long-oligonucleotide microarrays. BMC Genomics 2006;7:59.

2. Muthusamy V, Duraisamy S, Bradbury CM et al. Epigenetic silencing of novel tumor suppressors in malignant melanoma. Cancer Res 2006;66:11187-93.

3. Shames DS, Girard L, Gao B et al. A genome-wide screen for promoter methylation in lung cancer identifies novel methylation markers for multiple malignancies. PLoS Med 2006;3:e486.
